# Reproductive and Sexual Predictors of Late-Stage Cervical Cancer Presentation in Lagos, Nigeria

**DOI:** 10.7759/cureus.81934

**Published:** 2025-04-09

**Authors:** Adeyemi A Okunowo, Chinedu C Anumni, Muhammad Y Habeebu, Oluwatoyin M Olayioye

**Affiliations:** 1 Obstetrics and Gynaecology, College of Medicine, University of Lagos/Lagos University Teaching Hospital, Lagos, NGA; 2 Obstetrics and Gynaecology, Lagos University Teaching Hospital, Lagos, NGA; 3 Radiation Oncology, College of Medicine, University of Lagos/Lagos University Teaching Hospital/NSIA-LUTH Cancer Centre (NLCC), Lagos, NGA

**Keywords:** cervical cancer, lagos, late-stage presentation, menopause, nigeria, predictors, reproductive factors, sexual factors

## Abstract

Background

The impact of late-stage cervical cancer (CC) is huge in Nigeria and Sub-Saharan Africa. Several factors have been attributed to this late-stage presentation but studies that examined the contributory role reproductive and sexual factors play in the late-stage CC presentation are uncommon.

Objectives

This study aimed to identify reproductive and sexual factors contributing to the late-stage presentation of CC in Lagos, Nigeria.

Materials and methods

Records of 228 women with CC who presented to Lagos University Teaching Hospital between 2015 and 2019 were retrospectively reviewed and information on their reproductive, sexual, and clinicopathological characteristics was obtained for analysis. Data analysis was done using Statistical Package for Social Sciences (SPSS) version 23.

Results

Most (82.9%) of the women with CC presented with a late-stage disease with the majority presenting at stage 2B (28.9%) and stage 3A (20.2%) diseases. Among all the reproductive and sexual factors examined, only being post-menopausal was significantly associated with the increased likelihood of presenting with late-stage CC (crude odd ratio (COR)=2.26, confidence interval (CI)=1.13-4.55, p=0.020 and adjusted odd ratio (AOR)=2.15, CI=1.05-4.38, p=0.035). Age of menopause (AOR=2.08. CI=0.74-5.85, p=0.166) and duration of menopause (AOR=0.14, CI=0.16-1.30, p=0.143) were not significantly associated with late-stage CC presentation. Similarly, total pregnancies conceived (COR=1.32. CI=0.63-2.76, p=0.464), parity (AOR=1.68. CI=0.82-3.42, p=0.157), total children alive (COR=1.34. CI=0.67-2.68, p=0.413), presence of polygamous marriage (COR=1.52. CI=0.50-4.62, p=0.456), total lifetime sexual partners (COR=1.48. CI=0.66-3.31, p=0.342), age at coitarche (AOR=2.21. CI=0.68-7.12, p=0.186), and first delivery (AOR=2.71. CI=0.31-23.72, p=0.367) did not significantly influence late-stage CC presentation.

Conclusion

Menopausal status of the woman was the only reproductive and sexual factor that significantly predicted the likelihood of late-stage CC presentation with post-menopausal women having an increased risk of presenting with late-stage disease. There is a need for interventions to increase CC screening rates and improve health awareness and education about CC among post-menopausal women in Nigeria.

## Introduction

According to the GLOBOCAN 2022 cancer report, cervical cancer (CC) remains the most common female genital tract cancer and the 2nd most common female cancer worldwide [1], despite decades of availability of effective prevention, screening, and treatment strategies. Low- and middle-income countries (LMICs) contribute disproportionally to the high burden of the disease, with Sub-Saharan Africa (SSA) having the highest age-standardized (world) incidence and mortality rates [1]. Nigeria was the largest contributor to the number of new cases and deaths attributed to CC in the SSA region in 2022, accounting for 11.6% [2] and 9.3% [3] of the total numbers respectively. About 13,700 new cases of CC and 7,100 CC-related deaths were reported in Nigeria, making CC the 3rd most common cancer and cause of cancer-related mortality in the country respectively, after breast and prostate cancers [4].

Stage at presentation is critical in determining the management and health outcomes of cancer patients. Early-stage cancers are generally associated with better survival, reduced risk of cancer morbidities and mortality, and overall improved health outcomes compared to late-stage cancers [5]. Globally, approximately 60% of all CC cases present at a late stage, mainly due to contributions from LMICs [6]. While over 75% of the cases present with early disease in developed countries, a similar proportion present with late-stage in SSA [7]. Many women in Nigeria [7,8] and other African countries [9-10] often present with late-stage CC which is not ameliorable to cure but palliative treatment. It has been reported that out of the 34 per 100,000 new cases of CC that occur in Africa, 23 of the 100,000 women die of CC annually. This is in contrast to only 3 per 100,000 women dying of CC out of the 7 per 100,000 women being diagnosed in North America [9]. This disparity in incidence and mortality is largely due to the huge cases of late-stage CC presentation in the sub-region.

The impact of late-stage CC is vast and enormous in terms of its health, economic, and social outcomes. Women with locally advanced or metastatic CC frequently present with morbidities and cancer-related complications that limit treatment options and negatively impact health outcomes [9]. These include common complications like anemia, sepsis, obstructive uropathy with renal impairment, fistulous complications, and immunosuppression especially in the setting of HIV/AIDS [8]. These women are usually either unwell and unfit for treatment or are too poor to afford the cost of treatment. In addition, many LMICs lack or have limited radiotherapy facility services to cater to the huge cases of advanced CC leading to frequent delays or inability to access appropriate cancer treatment and ultimately poor health outcomes and survival. Furthermore, a significant proportion of the women with CC are in the reproductive age group who contribute significantly to the economic and social well-being of their family and community. Late-stage CC results in a significant loss of working hours, a reduction in productivity, loss of employment and economic power [11]. In the absence of health insurance coverage, as is usually the case in many SSA countries, the huge direct [12] and indirect costs [12] of treatment are borne by the woman and her family alone leading to severe financial toxicity, worsening of poverty, possible discontinuation of care, and adverse health outcomes [11]. Social alienation, discrimination, marital disharmony, and sexual dysfunction are a few of the reported social consequences associated with late-stage CC with a significant negative impact on quality of life [11].

Several factors have been reported to be associated with late-stage presentation of CC. These factors include poor awareness about the disease and screening tests [13], lack of organized screening programs [14], and poor uptake of the available screening services [10,14]. Some demographic and socio-economic factors such as age [14], education level [6], place of residence [6], income level [13], marital status, and financial constraints [10,14] have also been reported to be associated with late-stage diagnosis of CC. While some studies have reported on some reproductive and sexual characteristics of women with late-stage CC, only a little evidence exists on the in-depth conceptual analysis of reproductive and sexual factors as they relate to late-stage CC [15]. Furthermore, no peer-reviewed research in the literature has investigated the impact of sexual and reproductive factors on late-stage CC presentation in Nigeria, the largest contributor to CC incidence and mortality in the SSA region. This study sought to address existing knowledge gaps by thoroughly evaluating the reproductive and sexual factors linked to late-stage CC presentation in Lagos, Nigeria. Findings will inform targeted public health interventions and CC control strategies in Nigeria and the broader sub-Saharan African region.

## Materials and methods

Study design and setting

This was a retrospective cross-sectional study of women with histological diagnosis of CC who attended the Lagos University Teaching Hospital (LUTH), Lagos, Nigeria. LUTH is the largest tertiary hospital in Lagos, Southwestern Nigeria with more than 760-bed capacity and it caters to about 25 million people living in the state [16] and its environs. It is the state’s main referral center for oncology care and offers surgical and non-surgical cancer care services including radiation therapy, chemotherapy, and palliative care.

Study population and eligibility criteria

This included all women diagnosed with CC who received care in the institution between January 1st, 2015, and December 31st, 2019. Women who had histologically confirmed CC during the study period and complete data were included in the study while those without histological confirmation of CC, whose data could not be accessed, or who had incomplete data were excluded.

Data collection

The records of all women who attended the hospital’s Gynae-oncology and Radiotherapy outpatient clinics; Gynaecological Emergency unit, or admitted into the female in-lying wards during the study period were accessed and reviewed. Records of women with CC diagnosis were identified and meticulously retrieved from the medical records department of the Gynecology outpatient and Radiotherapy clinics. Information on the reproductive, sexual, and clinicopathological characteristics of eligible participants was obtained using a designed structured proforma. Reproductive characteristics of interest included the number of pregnancies ever conceived, parity, number of children alive, age at menarche, menstrual status, age, and duration of menopause, if menopausal. Sexual characteristics included age at coitarche, marriage, and first delivery; number of lifetime sexual partners, having partner with other sexual partners, history of polygamous marriage, sexually transmitted infections (STIs), and use of oral contraceptive pills (OCP). Information on the stage and histological type of CC was also retrieved. The disease staging was based on the International Federation of Gynecology and Obstetrics (FIGO) 2009 CC staging recommendation in use during the study period [17].

Data analysis

Study data was de-identified, cleaned, validated, and analyzed using Statistical Package for Social Sciences (SPSS) version 23.0, IBM Corp., Armonk, NY, USA. Descriptive statistics were computed and presented in tables or charts. Test for normality was done for continuous variables using the Shapiro-Wilk test. Normally distributed and skewed variables were expressed as mean ± standard deviation (SD) and median with interquartile range (IQR) respectively. The outcome variable was dichotomized into early and late-stage CC based on the outcome of the FIGO staging. Stage 2A and below were categorized as early stage while stage 2B and above were categorized as late-stage disease. Explanatory variables were grouped into categories and analysis was stratified based on these dichotomized outcomes and bivariate analysis was done using Pearson’s Chi-square test or Fischer’s exact test when the expected cell value was less than 5. Univariate regression analysis was done and crude odds ratios (OR) were computed. Variables with P-value <0.2 were entered into the multivariate regression analysis model and adjusted OR was computed. The level of statistical significance was set at a p-value < 0.05 at a 95% confidence interval.

Ethical consideration

Ethical approval (ADM/DCST/HREC/APP/3900) was obtained from LUTH’s Human Research and Ethical Committee before conducting the study. The study was carried out in accordance with the Declaration of Helsinki (1964).

## Results

A total of 426 gynecological cancers were managed in the institution during the study period, out of which 258 (60.6%) were histologically confirmed CC cases. Among these, only 228 (88.4%) CC cases had complete records and were included in the analysis.

Reproductive and sexual characteristics of women with cervical cancer

Table 1 shows the reproductive and sexual characteristics of women with CC during the study period. The median (IQR) number of pregnancies ever conceived and parity were 6 (4) and 5 (2) respectively with the majority, 139 (61.0%) and 125 (54.8%) having 5-9 pregnancies and childbirths respectively, while the median (IQR) number of children was 4 (3). The majority, 148 (64.9%) were post-menopausal with a mean ± SD age and duration of menopause of 48.9 ± 3.0 years and 13.4 ± 9.5 years respectively.

**Table 1:** Reproductive and sexual characteristics of participants *Among post-menopausal women n: total population; IQR: interquartile range; SD: standard deviation; STI: sexual transmitted infections The data has been represented as frequency (n), percentage (%), median (IQR), and mean ± SD.

Variable	Frequency (n = 228)	Percentage (%)
Pregnancy conceived		
0 – 4	65	28.5
5 – 9	139	61.0
> 10	24	10.5
Median (IQR) = 6 (4)		
Parity		
0 – 4	96	42.1
5 – 9	125	54.8
> 10	7	3.1
Median (IQR) = 5 (2)		
Number of children alive		
0 – 4	115	50.4
5 – 9	111	48.7
> 10	2	0.9
Median (IQR) = 4 (3)		
Age at menarche (years)		
<12	1	0.4
12 – 14	66	28.9
>14	161	70.6
Mean + SD = 15.3 + 1.8		
Menstrual status		
Pre-menopausal	80	35.1
Post-menopausal	148	64.9
*Age at menopause (years)	n = 148	
< 40	1	0.7
40 – 44	6	4.1
45 – 49	74	50.0
50 – 54	61	41.2
> 55	6	4.1
Mean + SD = 48.9 + 3.0		
*Duration of menopause (years)	n = 148	
1 – 10	72	48.6
11 – 20	47	31.8
21 – 30	24	16.2
>30	5	3.4
Mean + SD = 13.4 + 9.5		
Age at coitarche (years)		
<14	1	0.4
14 – 17	46	20.2
18 – 21	99	43.4
>21	82	36.0
Mean + SD = 19.9 + 2.9		
Age at marriage (years)		
15 – 19	33	14.5
20 – 24	168	73.7
25 – 29	26	11.4
≥30	1	0.4
Mean + SD = 22.0 + 2.4		
Age at first delivery (years)		
<20	21	9.2
20 – 24	122	53.5
25 – 29	77	33.8
≥30	8	3.5
Mean + SD = 23.5 + 3.3		
Number of lifetime sexual partners		
1	125	54.8
2	36	15.8
≥3	67	29.4
Median (IQR) = 1 (2)		
Partners having other sexual partners		
Yes	111	48.7
No	117	51.3
Polygamous marriage		
Yes	32	14.0
No	196	86.0
History of STI		
Yes	3	1.3
No	225	98.7
Use of oral contraceptive pills		
Yes	41	18.0
No	187	82.0

The mean ± SD age at sexual debut was 19.9 ± 2.9 with the majority, 99 (43.4%) and 82 (36.0%) having their first-ever sexual activity between 18 and 21 years and after 21 years, respectively. The median (IQR) number of lifetime sexual partners was 1 (2) with only 67 (29.4%) having three or more sexual partners. The majority, 168 (73.7%) and 122 (53.5%) got married and had their first childbirth between the ages of 20 and 24 years, respectively, and at a mean ± SD age at marriage and age at first delivery of 22.0 ± 2.4 years and 23.5 ± 3.3 years respectively. Even though 196 (86.0%) of the women were married in a monogamous relationship, many of their partners, 111 (48.7%) still had other sexual partners. Only 3 (1.3%) of the women had a history of STI and 41 (18.0%) used OCP.

Stage of cervical cancer at presentation

**Figure 1:**
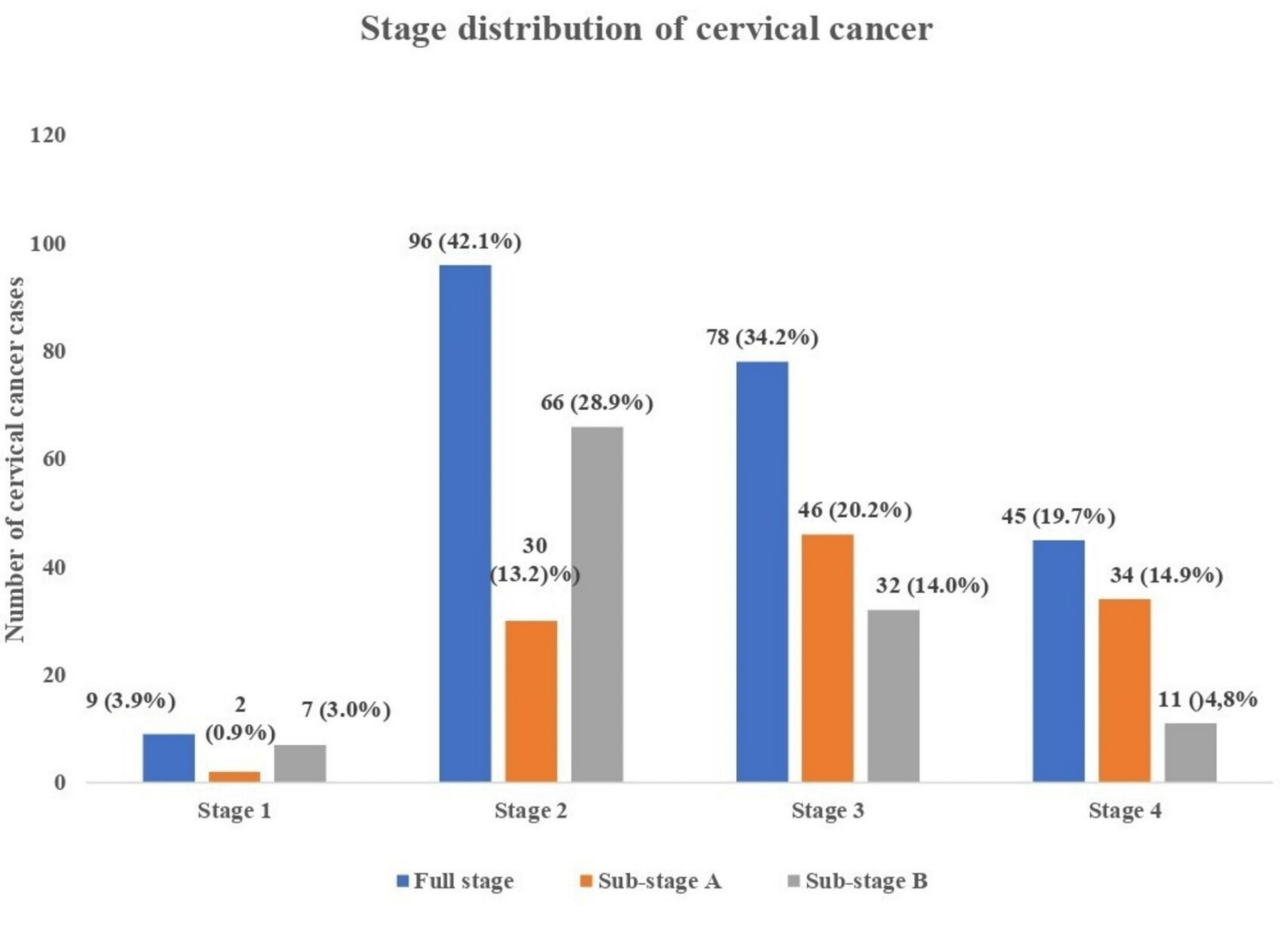
Stage distribution of cervical cancer cases The data has been represented as frequency (n) and percentage (%). Most of the women with cervical cancer presented in stage 2 (42.1%) and 3 (34.2%), specifically in stage 2B (28.9%) and 3A (20.2%), while only 3.9% presented in stage 1.

**Figure 2:**
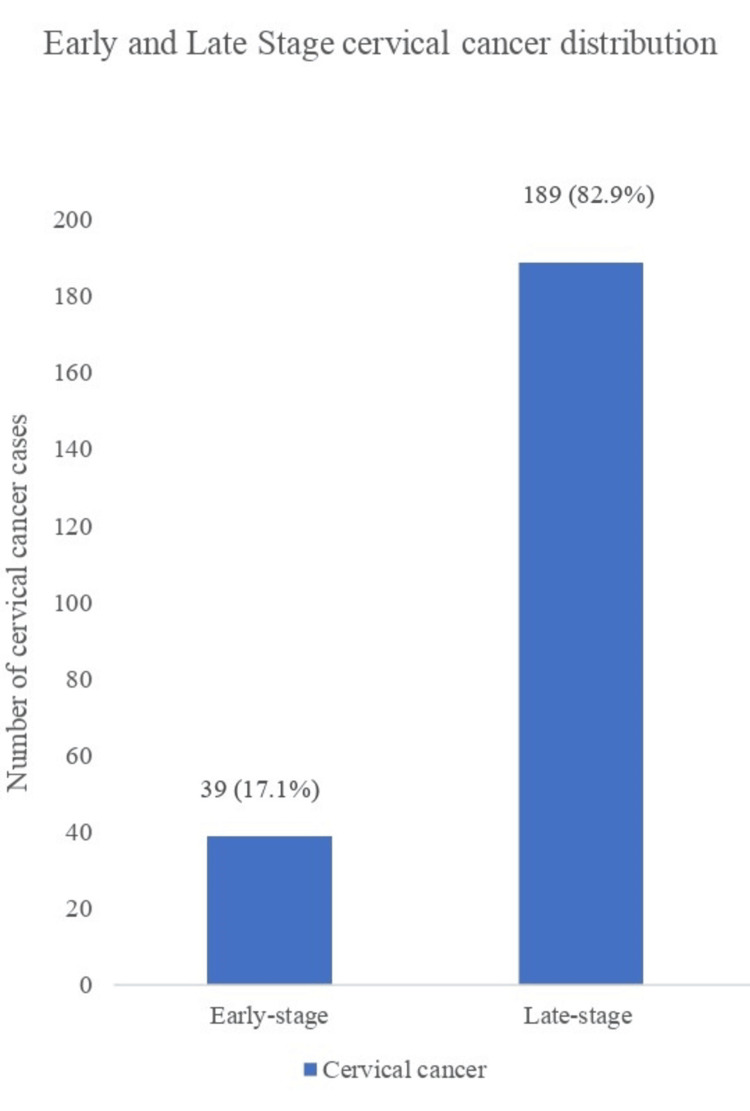
Early and late-stage cervical cancer distribution The data has been represented as frequency (n) and percentage (%) The majority (82.9%) of women with cervical cancer presented in late-stage while 17.1% presented early.

Figure 1 shows the stage distribution of CC at presentation. The most common stage at presentation was stage 2 with 96 (42.1%) women, followed by stage 3 and stage 4 diseases with 78 (34.2%) and 45 (19.7%) women, respectively. Stage 2B was the most common sub-stage of CC at presentation with 66 (28.9%) women, followed by sub-stage 3A and 4A with 46 (20.2%) and 34 (14.9%) women respectively. The majority, 189 (82.9%) of the cases of CC presented at late-stage disease, while only 39 (17.1%) presented at the early stage of the cancer (Figure 2). Most, 210 (92.1%) of the cancers were squamous in origin, while 14 (6.1%) were adenocarcinoma.

Reproductive and sexual factors associated with late-stage presentation of cervical cancer

Table 2 shows the association between reproductive and sexual factors and the stage at presentation of CC on bivariate analysis. Most of the reproductive and sexual factors did not show significant association with the stage at presentation except for the menstrual status of the participants (p = 0.020). The majority, 129 (68.3%) of women who presented with late-stage CC were predominately post-menopausal, compared to 20 (51.3%) women with early-stage CC who were mostly pre-menopausal. However, there was no significant association between the stage of CC presentation and age at menopause or duration of menopause (p = 0.269 and 0.256, respectively). Even though women with late stage of CC were of higher parity and had more children compared with those that presented at early stage disease, this was not statistically significant (p = 0.264 & 0.608, respectively).

**Table 2:** Reproductive and sexual factors associated with the stage of cervical cancer at presentation *Among post-menopausal women ^#^Fisher’s Exact test Statistical test = Pearson’s Chi-Square test n: total population; STI: sexually transmitted infections p-value <0.05 is considered significant. The data has been represented as frequency (n), and percentage (%).

	Stage of Cervical Cancer (%)		Chi-Square Test	
Variables	Early (n = 39)	Late (n = 139)		P
Pregnancy conceived			1.010	0.603
0 – 4	13 (33.3)	52 (27.5)		
5 – 9	21 (53.8)	118 (62.4)		
> 10	5 (12.8)	19 (10.1)		
Parity			2.663^#^	0.264
0 – 4	21 (53.8)	75 (39.7)		
5 – 9	17 (43.6)	108 (57.1)		
> 10	1 (2.6)	6 (3.2)		
Number of children alive			0.996^#^	0.608
0 – 4	22 (56.4)	93 (49.2)		
5 – 9	17 (43.6)	19 (49.7)		
> 10	0 (0.0)	2 (1.1)		
Age at menarche (years)			0.273^#^	0.872
<12	0 (0.0)	1 (0.5)		
12 – 14	12 (30.8)	54 (28.6)		
>14	27 (69.2)	134 (70.9)		
Menstrual status			5.417	0.020
Pre-menopausal	20 (51.3)	60 (31.7)		
Post-menopausal	19 (48.7)	129 (68.3)		
*Age at menopause (years)	n = 19	n = 129	3.934^#^	0.269
< 40	0 (0.0)	1 (0.8)		
40 – 44	1 (5.3)	5 (3.9)		
45 – 49	13 (68.4)	61 (47.3)		
50 – 54	4 (21.1)	57 (44.2)		
> 55	1 (5.3)	5 (3.9)		
*Duration of menopause (years)	n = 19	n = 129	4.148^#^	0.256
1 – 10	6 (31.6)	66 (51.2)		
11 – 20	8 (42.2)	39 (30.2)		
21 – 30	5 (26.3)	19 (4.7)		
>30	0 (0.0)	5 (3.9)		
Age at coitarche (years)			4.419^#^	0.220
<14	0 (0.0)	1 (0.5)		
14 – 17	4 (10.3)	42 (22.3)		
18 – 21	22 (56.4)	77 (40.7)		
>21	13 (33.3)	69 (36.5)		
Age at marriage (years)			6.411^#^	0.093
<20	7 (17.9)	26 (13.8)		
20 – 24	32 (82.1)	136 (72.0)		
≥25	0 (0.0)	27 (14.2)		
Age at first delivery (years)			2.745^#^	0.433
<20	1 (2.6)	20 (10.6)		
20 – 24	22 (56.4)	100 (52.9)		
25 – 29	14 (35.9)	63 (33.3)		
≥30	2 (5.1)	6 (3.2)		
Number of lifetime sexual partners			3.637	0.162
1	20 (51.3)	105 (55.6)		
2	10 (25.6)	26 (13.8)		
≥3	9 (23.1)	58 (30.6)		
Partners having other sexual partners			0.000	0.996
Yes	19 (48.7)	92 (48.7)		
No	20 (51.3)	97 (51.3)		
Polygamous marriage			0.557	0.456
Yes	4 (10.3)	28 (14.8)		
No	35 (89.7)	161 (85.2)		
History of STI			0.627^#^	0.428
Yes	0 (0.0)	3 (1.6)		
No	39 (100.0)	186 (98.4)		
Use of oral contraceptive pills			0.000	0.995
Yes	7 (17.9)	34 (18.0)		
No	32 (82.1)	155 (82.0)		

Additional analysis using univariate and multivariate regression models further supports the association between menopausal status and stage of CC presentation. On univariate analysis, being post-menopausal was associated with an increased odds of presenting with late-stage CC compared with pre-menopausal women (COR = 2.26, 1.13 - 4.55, p = 0.020). Though women who commence menopause at 48 years and above had increased odds of presenting with late-stage cancer, this was not statistically significant (COR = 2.20, CI = 0.79 - 6.14, p = 0.146). On the contrary, being menopausal for more than 10 years was associated with a 56% reduction in the odds of presenting with late-stage CC, however, this was also not statistically significant (COR = 0.44, CI = 0.16 - 1.23, p = 0.111). Though, having more than four pregnancies (COR = 1.32, CI = 0.63 - 2.76, p = 0.464), deliveries (COR = 1.78, CI = 0.89 - 3.55, p = 0.103) or children (COR = 1.34, CI = 0.67 - 2.68, p = 0.413), greater than two lifetime sexual partners (COR = 1.48, CI = 0.66 - 3.31, p = 0.342), being in a polygamous marriage relationship (COR = 1.52, CI = 0.50 - 4.62, p = 0.456) and starting sexual activities before 18 years (COR = 2.58, CI = 0.87 - 7.66, p = 0.079) were associated with increased odds of presenting with late-stage CC, these associations were not statistically significant. After adjusting for all relevant variables in a multivariate regression model, menopausal status was the only independent predictor of late presentation of CC (AOR = 2.15, CI = 1.05 - 4.38, p = 0.035) (Table 3).

**Table 3:** Reproductive and sexual predictors of late-stage cervical cancer presentation *Among post-menopausal women p-value <0.05 is considered significant.

Variables	Univariate			Multivariate			
	Crude Odds Ratio	P-value	95% Confidence Interval	Adjusted Odds Ratio	P-value	95% Confidence Interval	
Pregnancy conceived							
≤4	1			-			
>4	1.32	0.464	0.63 – 2.76	-			
Parity							
<5	1			1			
≥5	1.78	0.103	0.89 – 3.55	1.68	0.157	0.82 – 3.42	
Number of children alive							
<5	1			-			
≥5	1.34	0.413	0.67 – 2.68	-			
Age at menarche (years)							
≥15	1			-			
<15	0.93	0.835	0.44 – 1.95	-			
Menstrual status							
Pre-menopausal	1			1			
Post-menopausal	2.26	0.020	1.13 – 4.55	2.15	0.035	1.05 – 4.38	
*Age at menopause (years)							
<48	1			1			
≥48	2.20	0.146	0.79 – 6.14	2.08	0.166	0.74 - 5.85	
*Duration of menopause (years)							
≤10	1			1			
>10	0.44	0.111	0.16 – 1.23	0.14	0.143	0.16 – 1.30	
Age at coitarche (years)							
≥18	1			1			
<18	2.58	0.079	0.87 – 7.66	2.21	0.186	0.68 – 7.12	
Age at marriage (years)							
≥21	1			-			
<21	0.84	0.647	0.40 – 1.76	-			
Age at first delivery (years)							
>19	1			1			
≤19	4.50	0.138	0.59 – 34.55	2.71	0.367	0.31 – 23.72	
Number of lifetime sexual partners							
≤2	1			-			
>2	1.48	0.342	0.66 – 3.31	-			
Partners having other sexual partners							
No	1			-			
Yes	0.998	0.996	0.50 – 1.99	-			
Polygamous marriage							
No	1			-			
Yes	1.52	0.456	0.50 – 4.62	-			
History of STI							
No	-						
Yes	-						
Use of oral contraceptive pills							
No	1			-			
Yes	1.00	0.995	0.41 – 2.46	-			

## Discussion

Our study examined a population of women with histologically diagnosed CC over five years to determine the reproductive and sexual factors associated with the late-stage presentation of CC in Lagos, Nigeria. We found that more than three-quarters, 189 (82.9%) of the women with CC presented with late-stage disease with the majority, 66 (28.9%) presenting at stage 2B and 46 (20.2%) at stage 3A disease. We also found that though the number of pregnancies conceived, parity, number of children alive, total lifetime sexual partners, coitarche age, age at first delivery, menopausal status, age at menopause, and presence of polygamous marriage were associated with increased odds of a woman presenting with late-stage CC, only being post-menopausal was significantly associated with the increased likelihood of presenting with late-stage CC.

Our finding is consistent with the reports of late-stage CC presentation in many LMICs where more than 75% of women present with late-stage disease compared to the developed countries where more than 75% present in the early stage [7]. This is probably due to ineffective CC screening to detect early-stage CC in LMICs. A study on CC in 12 SSA countries by Burt et al reported that 85% of all new cases of CC present in late-stage disease [18]. At the country level, the proportion of women with late-stage CC presentation in our study is comparable to previous findings reported in other centers in Nigeria (83.0% - 89.3%) [7], Ethiopia (86.3%, 87.9%) [13,19], Uganda (80.0%) [20]. In Zaria, Nigeria [8], and Ghana [10], a staggering 98% and 95.2% respectively of all cases of CC presented in the late stage. Stage is an important determinant of survival and health outcomes in cancer management [6,7]. It is therefore not surprising that the highest mortality rates of CC are reported in SSA [1]. This may not be unconnected with the huge burden of late-stage presentation of the disease [21], bearing in mind that SSA has the highest burden of the disease globally [22].

Reproductive and sexual factors are well-established factors associated with the risk of developing CC but little is known about their impact on the stage of CC presentation. The focus has largely been on the influence of demographic and socio-economic factors on late presentation. Findings from our study showed that most of the reproductive and sexual factors examined did not significantly predict the likelihood of a woman presenting at a late stage with CC, except for menopausal status. This is contrary to the finding by Stewart et al. which found that high parity was associated with the risk of late-stage CC presentation [23]. Being menopausal independently predicted the risk of late-stage CC presentation. A woman who has CC and has attained menopause is predictably more likely to present with advanced CC compared to her counterpart who is yet to attain menopause.

Several reasons may be adjudged for the observed association between menopause and late-stage CC presentation. Menopause is a physiological landmark event in the life of a woman characterized by the cessation of the menstrual flow due to hormonal changes resulting in an increased risk of non-communicable diseases [24], including gynecologic cancers [25]. Post-menopausal women are a high-risk population for developing CC due to lack of adequate CC screening as the majority of these women exit the screening program early without meeting the recommended guideline criteria for cessation of screening [26]. Inadequate screening is a major risk factor for late-stage CC presentation [27] and the impact of this is more grievous in the SSA where the majority of women are either unscreened or under-screened [28], thus needing to commence or continue CC screening during menopause. Unfortunately, this is usually not the case as most post-menopausal women do not frequently engage in routine cervical screening. This practice is probably based on the false assumption that post-menopausal women are no longer at risk of developing CC and that screening is mainly for young sexually active women. Consequently, they do not see the need to engage in routine CC screening again and generally lack the motivation to do so. In addition, post-menopausal women are usually not the primary target of the few organized community or facility-based CC screening programs in our environment where the targeted population is mainly sexually active and reproductive-age women. These screening programs are missed opportunities to detect early-stage CC in this group of women. In our environment, menopause is usually associated with reduced sexual activities [29], and since the majority of these women are no longer sexually active, the incidence of postcoital bleeding which is an early manifestation of CC is usually absent. Even if this is present, this symptom may be normalized to be due to infrequent sexual activities. These factors are possible reasons why post-menopausal women are not only at increased risk of CC but also at increased risk of having late-stage disease.

Age is a critical factor in cancer development. The older a person is, the higher the risk of cancer development [30]. Post-menopausal women usually fall into the elderly age category where the risk of cancer is highest. This may partly explain why post-menopausal women are at increased risk of CC but may not explain why they present with late-stage disease. Abulajiang et al. investigated the influence of age at menopause on gynecological cancer risks and reported that early age at menopause significantly increased the risk of CC and other gynecological cancers [24]. Though this finding indirectly relates to the findings observed in our study, on the contrary, the duration of menopause or age at menopause did not significantly predict the risk of late-stage CC presentation in our study.

Limitations

Our study has some limitations that should be highlighted. Firstly, our study population was limited to a single institution which may not adequately represent the true characteristics of CC presentation in the real population. As a result, our findings cannot be generalized and should be interpreted in the context of the study setting. Secondly, the study outcome was limited only to the stage of CC at presentation. Even though stage is an important determinant of cancer outcome, our study did not assess the effect of the explanatory variables on other related outcomes such as treatment outcomes and survival. Being a retrospective study that reviewed medical records, the study is limited by incomplete or missing data, errors in documentation, recall bias, and inability to verify information collected. Finally, our study only examined the reproductive and sexual factors that influenced the late-stage presentation of women with CC. It did not assess the impact of other factors such as demographic and socio-economic factors and their confounding influence on the study explanatory variables in relationship to late-stage presentation. Further studies will be needed to evaluate this association.

Despite these limitations, our study is the first study to the best of our knowledge that comprehensively evaluated the impact of reproductive and sexual factors on late-stage CC presentation with the view of providing evidence that will guide future studies and targeted interventions that would reduce the burden of CC in Nigeria and by extension in the region.

## Conclusions

Our study reaffirms the high rate of late-stage CC presentation in the SSA region with more than three-quarters of the women with CC presenting late in Lagos, Nigeria, majorly at stage 2B and 3A disease. Among all the reproductive and sexual factors examined, menopausal status of the woman was the only factor that significantly predicted the likelihood of late-stage CC presentation with post-menopausal women having an increased risk of presenting with late-stage disease. There is a need for focused public health attention to increase the CC screening rates among women in the menopausal period to increase early detection and presentation of CC to improve health outcomes. In addition, there is a need to improve health awareness and education on CC among this group of women to encourage participation in the screening program and early presentation for care.
